# Differential Regulation of the Immune System in Peripheral Blood Following Ischemic Stroke

**DOI:** 10.1155/2022/2747043

**Published:** 2022-06-08

**Authors:** Wenhao Liu, Xin-Zhuang Yang, Dingding Zhang, Xin He, Qianlan Yu, Xinquan Liu, Yi Dai

**Affiliations:** ^1^Department of Neurology, Peking Union Medical College Hospital, Chinese Academy of Medical Sciences and Peking Union Medical College, Beijing, China; ^2^Medical Research Center, State Key Laboratory of Complex Severe and Rare Diseases, Peking Union Medical College Hospital, Chinese Academy of Medical Sciences and Peking Union Medical College, Beijing, China

## Abstract

**Method:**

108 IS samples and 47 matched controls were obtained from the GEO database. Immune-related genes (IRGs) and their associated drugs were collected from the ImmPort and PharmGBK databases, respectively. Random forest (RF) regression and least absolute shrinkage and selection operator (LASSO) logistic regression were applied to identify immune-related genetic biomarkers (IRGBs) of IS, and accuracy was verified using neural network models. Finally, proportion changes of various immune cells in peripheral blood of IS patients were evaluated using CIBERSORT and xCell and correlation analyses were performed between IRGBs and differentially distributed immune cells.

**Results:**

A total of 537 genes were differentially expressed between IS and control samples. Four immune-related differential expressed genes identified by regression analysis presented strong predictive power (AUC = 0.909) which we suggeseted them as immune-related genetic biomarkers (IRGBs). We also demonstrated six immune-related genes targeted by known drugs. In addition, post-IS immune system presented an increase in the proportion of innate immune cells and a decrease in adaptive immune cells in the peripheral circulation, and IRGBs showing significance were associated with this process.

**Conclusion:**

The study identified *CARD11*, *ICAM2*, *VIM*, and *CD19* as immune-related genetic biomarkers of IS. Six immune-related DEGs targeted by known drugs were found and provide new candidate drug targets for modulating the post-IS immune system. The innate immune cells and adaptive immune cells are diversified in the post-IS immune system, and IRGBs might play important role during this process.

## 1. Introduction

Stroke is the second leading cause of death and the main cause of disability among adults worldwide [1]. When an acute stroke (particularly ischemic stroke (IS)) is followed by a severe inflammatory attack, immune cells (such as microglia) inside the central nervous system (CNS) are activated and exert macrophage-like effects [2, 3]. More importantly, as the blood-brain barrier is disrupted, immune cells in the peripheral blood circulation can invade the CNS [4]. Proinflammatory signals from immune-mediators not only activate immune cells in situ but also recruit inflammatory cells (neutrophils, monocytes, macrophages, various types of T cells, and other inflammatory cells) from the peripheral blood to the ischemic area and induce a systemic inflammatory response [5]. Conversely, this early activation of the immune system is quickly replaced by a state of systemic immunosuppression that predisposes individuals to poststroke infections [4, 6]. In patients with stroke, the incidence of infectious complications, pneumonia, urinary tract infections, and other organ system infections is approximately 30%, and these symptoms are strongly associated with death and high recurrence rates in stroke survivors [7, 8].

Although changes in the immune system are relevant to the occurrence and development of IS, the pathogenesis underlying their variation is still unclear. Previous studies have reported that damaged brains can remodel peripheral immunity and inhibit the function of the peripheral immune system, leading to lymphopenia, reduced levels of inflammatory cytokines, and secondary lymphoid organ atrophy [3, 4, 9, 10]. The CNS can cause variations in immune function through complex humoral and neural pathways, including the sympathetic nervous system, vagus nerve, and hypothalamic-pituitary-adrenal (HPA) axis [9]. However, it remains unclear which signals and mechanisms trigger the sympathetic nervous system and the HPA axis to regulate the immune system following IS [6]. Comprehensive elucidation of the alteration in each component of the poststroke immune system is lacking, and studies on the molecular mechanisms underlying the changes are limited.

In this study, we identified immune-related genes whose expression highly associated with ischemic stroke and analyzed the correlation between these genes and different components of immune cells, in an attempt to understand the molecular mechanisms of immune system changes following IS and provide more evidence for the development of drugs for immune response in the future.

## 2. Materials and Methods

### 2.1. Microarray Data and Clinical Information

The microarray datasets used in this study were obtained from the GEO database (http://www.ncbi.nlm.nih.gov/geo/). The criteria for retrieving the datasets were (A) human peripheral whole blood samples, (B) all case samples were collected within acute phase of ischemic stroke, (C) gene expression profiling, (D) datasets containing both patients and healthy people without a history of stroke, where all patients were clinically diagnosed with ischemic stroke using medical imaging techniques (magnetic resonance imaging or X-ray computed tomography), and (E) datasets of patients with hemorrhagic stroke were excluded.

To ensure consistency and completeness of the datasets, we manually identified relevant literature using keyword filters. Finally, two datasets (GSE16561 [11–13] and GSE58294 [14]) were included and treated as “traning set.” These datasets were merged, and batch-effects between different datasets were corrected using the “combat” function in the SVA package (version: 3.38.0) [11]. Next, we normalized the merged dataset and completed covariate adjustment, using the “Normalizebetweenarrays”and “removeBatchEffect” function in the limma package (version: 3.46.0) [15, 16]. For validation of biomarker genes, we downloaded GSE22255 [17], GSE37587 [18], and GSE46480 [19] datasets which satisfied most criterions while only containing IS or normal cohorts (Table 1).

### 2.2. Identification of Differential Expressed Genes and Functional Annotation

To identify differentially expressed genes (DEGs) in peripheral blood samples from ischemic stroke patients and controls, we performed differential expression analysis using the limma package (version: 3.46.0), controlling for age. The threshold for screening DEGs was |log2 FC (fold change)| > 0.5 and FDR < 0.001. Enrichment analysis of Gene Ontology (GO) and Disease Ontology (DO) was performed on DEGs using the clusterprofiler package (version: 3.18.1) [20]. Reference to the Kyoto Encyclopedia of Genes and Genomes (KEGG) database (http://www.genome.jp/kegg/) and gene set enrichment analysis (GSEA) was carried out on the gene expression matrix [21, 22]. The significance of KEGG signaling pathways was set at FDR < 0.05.

### 2.3. Immune-Related Genes and Drug Targets

Immune-related genes (IRGs) and “variant, gene, and drug relationship” datasets were downloaded from the ImmPort database (https://www.immport.org/shared/genelists) and PharmGBK database (https://www.pharmgkb.org/downloads), respectively [23, 24]. The intersection of DEGs, IRGs, and drug target genes (DTGs) was then used to generate immune-related DEGs targeted by drugs and potential drugs that may contribute to the changes in the post-IS immune system.

### 2.4. Selection and Validation of Immune-Related Genetic Biomarkers

Four IRGBs in IS, namely, *CARD11*, *ICAM2*, *VIM*, and *CD19*, were identified from the immune-related DEGs using LASSO logistic regression and random forest (RF) regression algorithms with training datasets. The LASSO algorithm was derived from the glmnet package (version: 4.1-1) [25]. The neural network was built using tensorflow2 framework (version: 2.3.0) using Python language and based on the merged dataset [26, 27]. GSE37587 and GSE46480 were used as test sets to verify the sensitivity and efficiency of IRGBs in IS diagnosis. Although the data sample is derived from peripheral blood mononuclear cells (PBMCs), GSE22255 was also used as an independent test set for additional validation.

### 2.5. Immune Cell Infiltration Evaluation

CIBERSORT immune system analysis tool and the deconvolute_Xcell function in the immunedeconv package (version: 2.0.4) were used to generate immune cell composition profiles for all samples [28, 29]. The CIBERSORT immune system analysis tool results in an expression matrix of 22 immune cells in all samples of the merged dataset, while the deconvolute_Xcell function results in an expression matrix of 67 immune-related variables versus all samples. We then used *t*-test to analyze the differences in immune cell components between IS patients and healthy people. Finally, Spearman's correlation analysis was performed between IRGBs and significantly differentiated immune cells using the ggstatsplot package (version: 0.7.2). The ggplot2 package (version: 3.3.3) was used to generate visual heatmaps [30].

## 3. Result

### 3.1. DEGs Enriched in Immune-Related Biological Process

**Figure 1:**
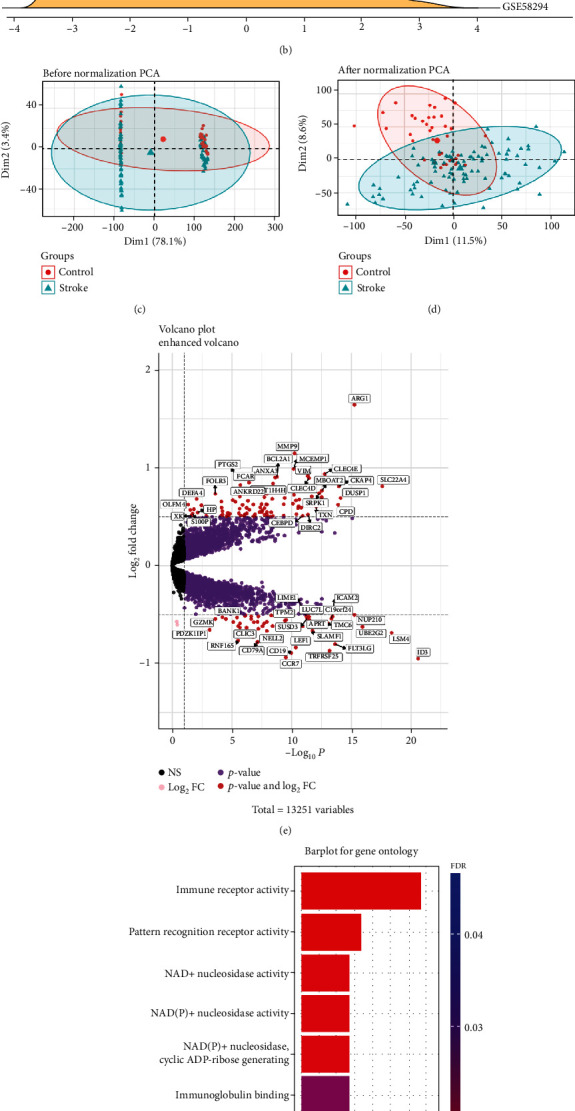
(a) The ridge plot of the merged dataset before eliminating the batch effect. (b) The ridge plot of the merged dataset after eliminating the batch effect. (c) The Principal Component Analysis (PCA) plot of the merged dataset before normalization. (d) The PCA plot of the stroke group and control group of the merged dataset after normalization. Note: In PCA analysis, all genes were used to observe the effect of normalization. (e) Volcano map of DEGs; red represents significant differential genes, grey represents no significant difference genes, pink represents genes with differential log_2_FC, and purple represents genes with differential *P* value. (f) GO enrichment analysis, where the horizontal axis represents the number of DEGs under the GO term.

We first generated an independent dataset of 155 samples consisting of 108 IS patients and 47 matched controls, by merging two datasets: GSE16561 and GSE58294 (Tables 1 and 2, Materials and Methods). To ensure data consistency, batch-effect was controlled and the different subsets were normalized. The results showed that data preprocessing was effective and reliable (Figures 1(a)–1(d), Figure S1). Next, we performed differential analysis of gene expression by controlling age and 537 DEGs between IS patients and healthy controls were identified. (Figure 1(e)).

GO enrichment analysis showed that DEGs were mainly associated with immune receptor activation, pattern recognition receptor activation, and NAD^+^ nucleotidase activation (Figure 1(f)), while DO enrichment analysis showed that the diseases enriched within DEGs included mainly arteriosclerosis, atherosclerosis, arteriosclerotic cardiovascular disease, myocardial infarction, obesity, nutritional diseases, and coronary artery disease (Figure S2A). These diseases are intimately associated with IS, indicating that the DEGs are involved in the occurrence and development of IS.

Furthermore, the GSEA results showed that the molecular pathways enriched with DEGs were complement and coagulation cascades, neutrophil extracellular trap formation (NETs), lipid and atherosclerosis, tumor necrosis factor (TNF) signaling pathway, Toll-like receptor signaling pathway, and NOD-like receptor signaling pathway (Figure S2B). Of these, the Toll-like receptor signaling pathway, NOD-like receptor signaling pathway, and TNF-*α* signaling pathways play important roles in innate immunity (Figure S2C), consistent with the results of the GO enrichment analysis. These results provide evidence that immune-related biological processes might play important roles in IS.

### 3.2. Four Immune-Related Genetic Biomarkers Perform Well in IS Diagnosis

**Figure 2:**
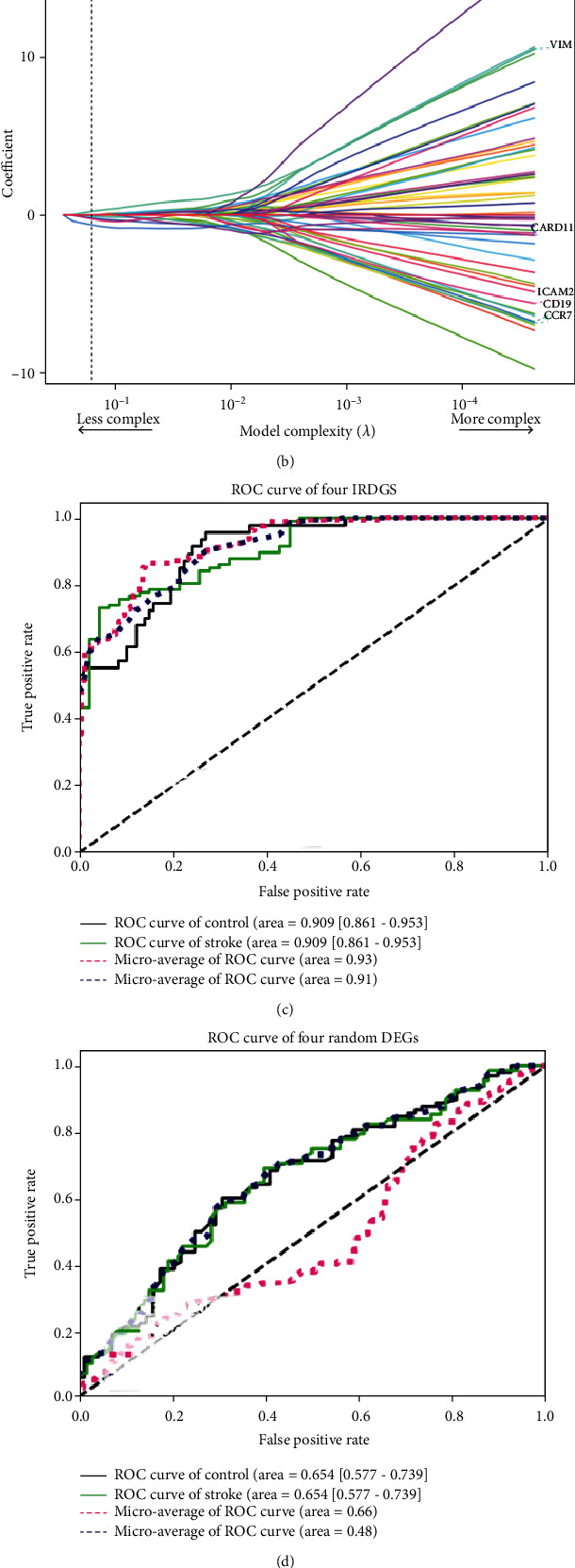
(a) and (b) Construction of the IRG diagnostic classifier by the LASSO logistic regression algorithm shows the process of dimension reduction and coefficient decomposition. The horizontal axis represents the complexity of models, and the vertical axis represents the AUC value of models and model coefficient of labeled gene expression, respectively. (c) Construction of the IRG diagnostic classifier by the random forest regression algorithm. (d) The ROC curve of the neural network diagnosis model using four selected diagnostic markers. (e) The ROC curve of the neural network diagnosis model using four random DEGs.

To further determine which kinds of immune genes are dramatically altered and associated with the occurrence of IS, we performed LASSO logistic regression analysis on immune-related DEGs generated from the intersection of IRGs and DEGs and found five genetic markers in peripheral blood (Figure 2(a), Materials and Methods). Among them, *VIM* was positively correlated with the occurrence of stroke, while *CARD11*, *ICAM2*, *CD19*, and *CCR7* were negatively correlated with the occurrence of stroke (Figure 2(b)). Meanwhile, ten genetic markers that reached significance were discovered using the RF algorithm (Figure 2(c), Materials and Methods). Surprisingly, four genes, *VIM*, *CARD11*, *ICAM2*, and *CD19,* were identified by both methods and we suggested them as immune-related genetic biomarkers (IRGBs) which might be used for auxiliary diagnosis of the disease. In order to eliminate the effect of age difference between the two groups, we controlled participants' age during analysis. The results demonstrated that gene expression of IRGBs showed no correlation with age and confirmed that our findings are truly between patients and healthy controls (Figure S3).To further verify the sensitivity and accuracy of these four genes in IS diagnosis, GSE37587 and GSE46480 were used as validation datasets, and a neural network model was used to examine the prediction performance (Figure S4). The results were excellent (AUC = 0.909; 95%CI = [0.861, 0.953], Figure 2(d)). We also randomly selected four DEGs and performed the same analysis. The prediction performance of these randomly selected DEGs was poor (AUC = 0.654;95%CI = [0.577, 0.739], Figure 2(e)), demonstrating that the our IRGBs were significantly associated with the occurrence of IS. To further verify the conclusion, we select another dataset whose samples derived from PBMCs, and the diagnostic ability of the IRGBs is still good (AUC = 0.835; 95%CI = [0.701, 0.951], Figure S5).

**Figure 3:**
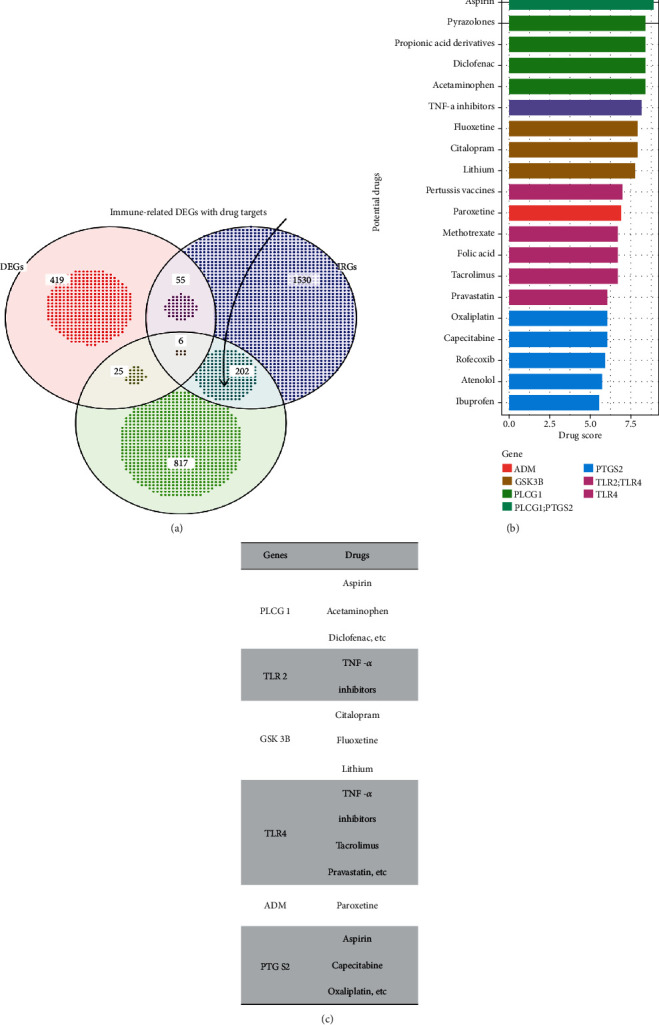
(a) Venn diagram shows the target genes of potential drugs obtained by the their gene sets. (b) and (c) Barplot and table show potential drugs and their targeted genes.

Considering that immune-related genes present significant changes after the onset of stroke (Figure 1(f)), we sought to explore whether there are drugs that can mitigate the process. We identified overlapping genes across three gene sets, DEGs, IRGs, and DTGs, and found six immune-related DEGs with known drug targets, including *PLCG1*, *TLR2*, *GSK3B*, *TLR4*, *ADM*, and *PTGS2* (Figure 3(a)). Drugs targeting these six genes include aspirin, pyrazolone (e.g., phenylbutazone), propionate derivatives (e.g., ibuprofen), diclofenac, paracetamol, and TNF-*α* inhibitors such as pravastatin (Figures 3(b) and 3(c)). Aspirin is already recommended for early treatment of stroke. In addition to acting as an antiplatelet, it plays an important role in modulating the post-IS immune environment [31]. TLR2 and TLR4 are pattern recognition receptors (Figure 3(b)) and reveal the pattern recognition function of the Toll-like receptor signaling pathway in regulating the post-IS immune system.

### 3.3. IRGBs Are Associated with the Change of Immune System following IS

**Figure 4:**
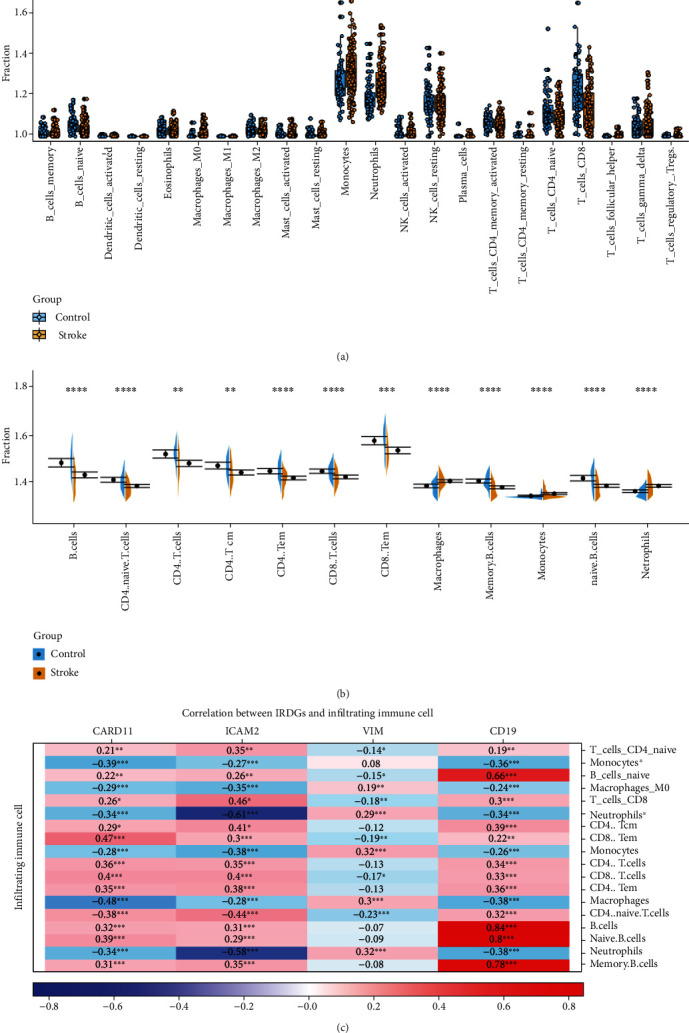
(a) and (b) Violin diagram of the proportion of immune cells obtained by CIBERSORT and xcell tool. The yellow marks represent the difference in immune cell proportion between the two groups of samples. ^∗^*P* < 0.05; ^∗∗^*P* < 0.01; ^∗∗∗^*P* < 0.001; ^∗∗∗∗^*P* < 0.0001. (c) Correlation between *CARD11*, *ICAM2*, *VIM*, *CD19*, and significant deferential immune cells. ^∗^: Monocytes^∗^ and neutrophils^∗^ were generated by CIBERSORT tool. ^∗^*P* < 0.05; ^∗∗^*P* < 0.01; ^∗∗∗^*P* < 0.001; ^∗∗∗∗^*P* < 0.0001.

We applied the CIBERSORT classification algorithm to the merged dataset to demonstrate changes of the immune system after IS. Interestingly, the proportion of naive B cells, M0-type macrophages, monocytes, neutrophils, CD8^+^ T cells, and CD4^+^ naive T cells was significantly different between IS patients and healthy controls (Figure 4(a)). M0-type macrophages and monocytes were significantly enriched in the peripheral blood of IS patients, while naive B cells, CD8^+^ T cells, and naive CD4^+^ T cells were significantly decreased. In addition, using the xCell deconvolution algorithm to compute enrichment scores for 67 immune-related factors, we found that 12 immune cells represented significantly different constituent components between IS patients and healthy controls (Figure 4(b)). More specifically, neutrophils, macrophages, and monocytes were significantly enriched in the peripheral blood of IS patients compared with healthy controls, consistent with the results of the CIBERSORT classification algorithm. Additionally, there were 9 immune cells with enrichment fractions significantly lower than in healthy controls, including multiple types of B cells and T cells.

Correlation analysis indicated a close relationship between IRGBs and 18 differentially distributed immune cells (Figure 4(c)). *CARD11* was positively correlated with CD8^+^ effective memory T cells (*r* = 0.47, *P* < 0.001, Figure 4(c)) and negatively correlated with macrophages (*r* = −0.48, *P* < 0.001); *ICAM2* was positively correlated with CD8^+^ T cells (*r* = 0.46, *P* < 0.001) and negatively correlated with neutrophils (*r* = 0.61, *P* < 0.001); *VIM* was slightly correlated with neutrophils (*r* = 0.32, *P* < 0.001) and macrophages (*r* = 0.3, *P* < 0.001); and *CD19* was positively correlated with B cells (*r* = 0.84, *P* < 0.001), naive B cells (*r* = 0.8, *P* < 0.001), and memory B cells (*r* = 0.78, *P* < 0.001). Our analysis showed that IRGBs are significantly associated with changes in the immune environment after IS, demonstrating an important role in IS diagnosis and therapy.

## 4. Discussion

Acute stroke (mainly IS) is followed by severe inflammatory episodes. With the blood-brain barrier being disrupted, immune cells in the peripheral circulation invade the CNS. Many studies have demonstrated that neutrophils, monocytes, macrophages, and various types of T cells in the peripheral blood infiltrate the ischemic area and induce systemic inflammatory responses [32–35]. However, this early activation of the immune system is present only for a short period and is generally replaced by a state of systemic immunosuppression that predisposes one to poststroke infection rapidly after stroke. Both morbidity and mortality due to infectious diseases are much higher in IS patients than in the normal population, and the alteration in the immune system is closely associated with a high recurrence rate in stroke survivors [7, 8].

In this study, 537 DEGs were found between IS patients and healthy controls. Four significantly differential expressed immune-related genes, namely, *CARD11*, *ICAM2*, *VIM*, and *CD19*, were identified by both LASSO logistic regression and RF algorithm analysis. Furthermore, these four genes were used to construct a neural network prediction model and presented excellent predictive value and accuracy in validation datasets.

CARD11 is a scaffold protein composed of 1154 amino acids and belongs to the membrane-associated guanylate kinase (MAGUK) superfamily [36]. *CARD11* is expressed in peripheral leukocytes and is an important component of the antigen inducible NF-*κ*B signaling pathway in T cells [37]. Intercellular adhesion molecule-2 (ICAM2) belongs to the ICAM family of adhesion proteins [38]. It is widely expressed in vascular endothelial cells and peripheral blood cells and plays a key role in cell-cell interactions, promoting neutrophil binding to, and transmigration across, the vascular endothelium [39]. *ICAM2* is also a key gene in platelet leukocyte interaction and participates in IS by regulating the platelet leukocyte aggregation process [40]. VIM protein (vimentin) is part of the intermediate membrane system, which plays an important role in maintaining cell shape, resisting mechanical stress, transmitting cytoskeletal crosstalk, and organizing signaling molecules [41]. Vimentin also has an important role in maintaining plasticity in the nervous system, and several studies have demonstrated that its expression is upregulated after ischemic stroke [42–45]. *CD19* is widely expressed during B cell development and is a key coreceptor for B cell antigen receptor signal transduction [46]. Courties et al. showed that cerebral ischemic injury leads to an organismal stress response that activates the HPA axis and mediates B lymphopoiesis deficiency, which could explain the downregulated expression of *CD19* in the peripheral blood of IS patients [9]. The neural network prediction model exhibited excellent accuracy and sensitivity using these four IRGBs.

GO enrichment analysis showed that DEGs were mainly associated with immune receptor activation, pattern recognition receptor activation, and NAD^+^ nucleotidase activation. The diseases enriched by DO were mainly atherosclerosis, myocardial infarction, obesity, nutritional diseases, and coronary artery disease. The results of GO enrichment analysis indicated that the immune response plays an important role in IS, and the diseases enriched via DO are highly correlated with IS. Moreover, the pathways enriched in GSEA mainly involved complement and lectin cascades, NETs, lipid and atherosclerosis, TNF signaling pathway, Toll-like receptor signaling pathway, and NOD-like receptor signaling pathway. Previous studies have demonstrated that the signaling pathways of complement and coagulation cascades, lipids, and atherosclerosis are the core factors of IS [47–49]. Laridan et al. showed that neutrophils and NETs are important components of cerebral thrombi and are more abundant in thrombi for longer, suggesting that NETs play an important role in the progression of IS [50]. TNF-*α* is one of the typical proinflammatory cytokines. Cui et al. suggested that it plays an important role in the development of IS, and the activation of microglia induces TNF-*α* expression and activation and recruitment of circulating neutrophils, monocytes, and lymphocytes into the CNS [51]. Toll-like receptors, key pattern recognition receptors in innate immunity, are also crucial for NET formation by neutrophils as well as infiltration by monocytes, contributing to thrombus formation [52]. NOD-like receptor proteins containing nucleotide-binding oligomerization domains can recognize damage-associated molecular patterns in sterile inflammation [53]. Yang et al. found that NADPH oxidase-mediated NLRP3 signaling contributed to cerebral ischemic injury by aggravating inflammation and neurovascular injury [54]. The above findings were consistent with our analysis results.

In addition to differential expressed genes, six immune-related DEGs were found to be targeted by drugs: *PLCG1*, *TLR2*, *GSK3B*, *TLR4*, *ADM*, and *PTGS2*. Drugs targeting these six genes include aspirin, pyrazolone (e.g., phenylbutazone), propionate derivatives (e.g., ibuprofen), diclofenac, paracetamol, and the TNF-*α* inhibitor, pravastatin. Aspirin and statins, alone or in combination, have been shown to modulate the secretome profile of peripheral blood monocyte macrophages after stroke [55]. Toll-like receptor signaling pathway is important in mediating the formation of immune system after stroke, and previous animal experiments have shown that TLR2 or TLR4 deficient mice have less brain tissue damage after stroke than wild-type mice [56].

To further explore immune cell changes after IS, we performed immune cell enrichment analysis on the merged dataset using CIBERSORT and xCell tools. The results showed that the enrichment fractions of neutrophils, macrophages, and monocytes increased while the enrichment fractions of various types of B cells and T cells decreased, which may be due to the occurrence and development of IS. Previous studies have shown that neutrophils are one of the first blood-derived immune cells to enter and be stationed in brain tissue in most experimental stroke models [32, 33]. Weston et al. reported that neutrophil infiltration was elevated on the first day, peaked on the third day, and began to decline, but was still present at 7 and 15 days after cerebral ischemia, with the degree of increase positively correlated with infarct volume and functional deficits [34]. Kaito et al. found an increase in the total monocyte-macrophage numbers in the human peripheral blood circulation early after brain injury [32]. In the chronic phase of IS, 3–7 days after the onset of ischemia, monocyte-derived macrophages from the peripheral blood circulation peak at the injured site [4]. GöKhan et al. found that, in contrast to neutrophils, monocytes, and macrophages, patients with ischemic stroke have a reduced number of lymphocytes in the peripheral blood and consequently an increased neutrophil/lymphocyte ratio [33]. Previous studies have shown that after IS, B cell development in the bone marrow is severely impaired at the pro-B cell stage and that this impairment leads to peripheral lymphopenia [9]. During this period, there is evidence of a shift in CD4^+^ T cells from a Th1 type response mediated by cellular immunity to a Th2 type response mediated by humoral immunity, and this shift protects the brain from further damage [35]. However, the immune system is suppressed, and the number of T lymphocytes and B lymphocytes in the peripheral blood is reduced. The evidence from these studies combined with the results from our analysis suggests that neutrophils, macrophages, monocytes, various types of B cells, and T cells play important roles in IS and should be the focus of further research. There is currently a lack of in-depth research exploring the relationship between B cells and T cells in each category and the occurrence of IS, and further experiments are needed to reveal more details. Furthermore, our results revealed correlations between the four IRGBs and differentially expressed immune cells in IS. *CARD11* was significantly positively correlated with CD8^+^ effector memory T cells and negatively correlated with macrophages; *ICAM2* was significantly positively correlated with CD8^+^ T cells and negatively correlated with neutrophils; *VIM* was positively correlated with neutrophils and macrophages; and *CD19* was significantly positively correlated with B cells, naive B cells, and memory B cells. *CARD11* is ubiquitously expressed in peripheral leukocytes and is important in inducing the activation of NF-*κ*B signaling pathway in T cells, but its relationship with macrophages is currently unclear. CD8^+^ T cell-mediated immunosurveillance depends on the LFA-1: ICAM adhesion pathway for recognition, whereas ICAM2 is involved in neutrophil-mediated plasma leakage, perhaps explaining the association between *ICAM2* and CD8^+^ T cells and neutrophils [38, 39]. Multiple studies have shown that vimentin can activate ERK1/2 signaling to recruit macrophages and neutrophils [41–43]. *CD19* is widely expressed on almost all B cells, and their significant positive relationship justifies the analysis results. Research is still needed to clarify the relationship between IRGBs and immune cells.

In this study, RF algorithm and LASSO logistic regression algorithm were used to search for IS genetic biomarkers, and a neural network was built to verify the accuracy. RF is a nonparametric tree-based machine learning method that searches for optimal variables with minimum depth statistics [57]. LASSO logistic regression belongs to the shrinkage estimation, and during the reduction process of regression coefficients, some insignificant regression coefficients can be directly reduced to 0, that is, to the function of variable screening [58]. These two algorithms were used to screen the feature variables and construct the best classification model. In this study, *CARD11*, *ICAM2*, *VIM*, and *CD19* were identified as genetic biomarkers for IS by combining the RF and LASSO logistic regression methods. The biomarkers selected by integration performed excellently in the neural network prediction model.

The ImmPort and PharmGBK databases were applied, based on a comprehensive analysis of gene expression and drug activity, to select immune-related DEGs targeted by drugs and new drug targets that could modulate the post-IS immune system. To our knowledge, it is the first time that CIBERSORT and xCell have been used together, in the analysis of post-IS immune system changes in peripheral blood, and their performance has been verified to each other. CIBERSORT and xCell were both based on limited genetic data that may deviate from heterotypic interactions of cells, disease-induced disorders, or phenotypic plasticity. The information on immune genes and drug targets included in the ImmPort and PharmGBK databases was also incomplete. Furthermore, our study represents secondary mining and analysis of previously published datasets. Although several previous findings are consistent with our analysis, the reliability of the results of this study requires further experimental validation.

## Figures and Tables

**Table 1:** All datasets used in this study contain a total of 361 samples, among which there were 196 cases and 165 controls. All case samples were collected acute phase of ischemic stroke.

Datasets (GEO ID)	Data		Sample type/source	References	Category	GPL
	Case	Control				
GSE16561	39	24	Peripheral blood	(Barr et al., 2010; O'Connell et al., 2016; O'Connell et al., 2017) [11–13]	Train	GPL570
GSE58294	69	23	Peripheral blood	(Stamova et al., 2014) [14]	Train	GPL570
GSE37587	68	0	Peripheral blood	(Barr et al., 2015) [18]	Test	GPL6883
GSE46480	0	98	Peripheral blood	(Issa et al., 2016) [19]	Test	GPL570
GSE22255	20	20	PBMCs	(Krug et al., 2012) [17]	Test	GPL570

**Table 2:** Clinical characters of the merged data set.

	Total sample, *N*(%)	Stroke *N* = 62 (56.9%), *N*(%)	Control, N = 47 (43.1%), *N*(%)	Statistics/*df*	*P* value
Gender (% female)	58(53.2%)	33(53.2%)	25(53.2%)	X^2^ 0.000013/1	0.9972
Age, y, mean ± SD	66.7 ± 16.86	72.6 ± 12.09	58.9 ± 7.51	*t*-13.90302/107	<0.001
Race (% white)	97(89.0%)	54(87.1%)	43(91.5%)	X^2^ 0.526507/1	0.4681
Hypertension	64(58.7%)	41(66.1%)	23(48.9%)	X^2^ 3.26002/1	0.07099
Diabetes	22(20.2%)	15(24.2%)	7(14.9%)	X^2^ 1.43527/1	0.2309
Dyslipidemia	40(36.7%)	24(38.7%)	16(34.0%)	X^2^ 0.250672/1	0.6166

## Data Availability

The microarray data used to support the findings of this study have been deposited in the GEO database(GSE16561:https://www.ncbi.nlm.nih.gov/geo/query/acc.cgi?acc=GSE16561; GSE58294:https://www.ncbi.nlm.nih.gov/geo/query/acc.cgi?acc=GSE58294;GSE37587:https://www.ncbi.nlm.nih.gov/geo/query/acc.cgi?acc=GSE37587;GSE46480:https://www.ncbi.nlm.nih.gov/geo/query/acc.cgi?acc=GSE46480;GSE22255:https://www.ncbi.nlm.nih.gov/geo/query/acc.cgi?acc=GSE22255).

## Supplementary Materials

Supplementary Materials Supplement Figure 1: quantile-quantile (Q-Q) plots show that the interbatch differences among four selected datasets are eliminated. Gray shading represents the 95% confidence intervals around the normal distribution. Supplement Figure 2: (A) Disease ontology (DO) enrichment analysis. The size of the circle corresponds to the number of differentially expressed genes (DEGs) under the DO term. (B) Gene set enrichment analysis, where the horizontal axis represents up-and-down regulation and the curve area represents the number of DEGs under the gene set enrichment analysis (GSEA) term. (C) Gene set enrichment analysis. *P* values were determined using the Kolmogorov-Smirnov test. Supplement Figure 3: correlation analysis between gene expression of IRGBs and sample age. Supplement Figure 4: structure of the neural network. Supplement Figure 5: the ROC curve of the neural network diagnosis model using four selected genetic biomarkers in GSE22255 dataset.
