# Supplementary material for: Differential Regulation of the Immune System in Peripheral Blood Following Ischemic Stroke
Source: Biomed Res Int. 2022 Jun 8;2022:2747043. doi: 10.1155/2022/2747043 (PMC9200570; doi:10.1155/2022/2747043)
Supplement: Supplementary Materials — Supplement Figure 1: quantile-quantile (Q-Q) plots show that the interbatch differences among four selected datasets are eliminated. Gray shading represents the 95% confidence intervals around the normal distribution. Supplement Figure 2: (A) Disease ontology (DO) enrichment analysis. The size of the circle corresponds to the number of differentially expressed genes (DEGs) under the DO term. (B) Gene set enrichment analysis, where the horizontal axis represents up-and-down regulation and the curve area represents the number of DEGs under the gene set enrichment analysis (GSEA) term. (C) Gene set enrichment analysis. P values were determined using the Kolmogorov-Smirnov test. Supplement Figure 3: correlation analysis between gene expression of IRGBs and sample age. Supplement Figure 4: structure of the neural network. Supplement Figure 5: the ROC curve of the neural network diagnosis model using four selected genetic biomarkers in GSE22255 dataset. [file 2747043.f1.docx]

**
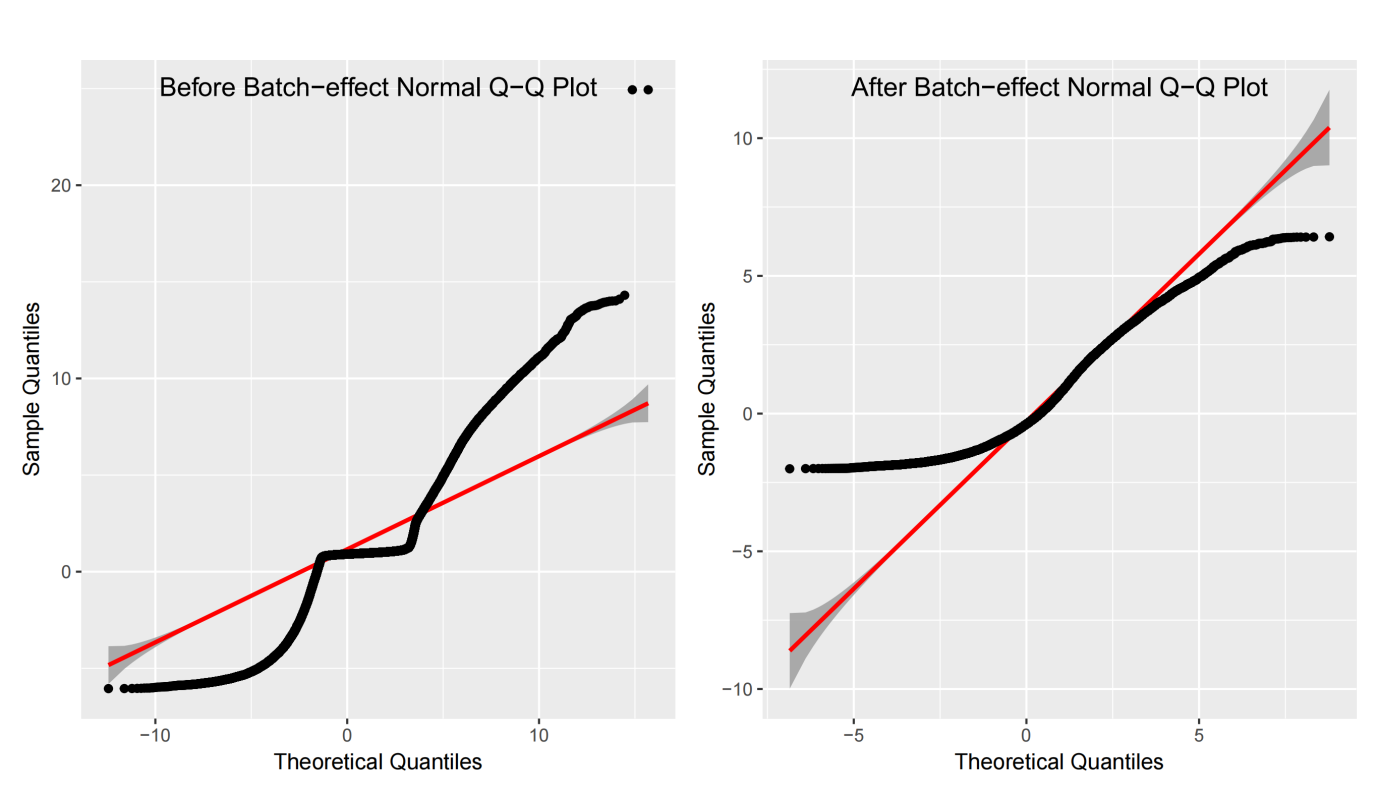
**

**Supplement Figure 1.** The quantile-quantile (Q-Q) plot shows the inter-batch difference of four selected datasets is removed. The shadow is the 95% confidence interval of the normal distribution.

0


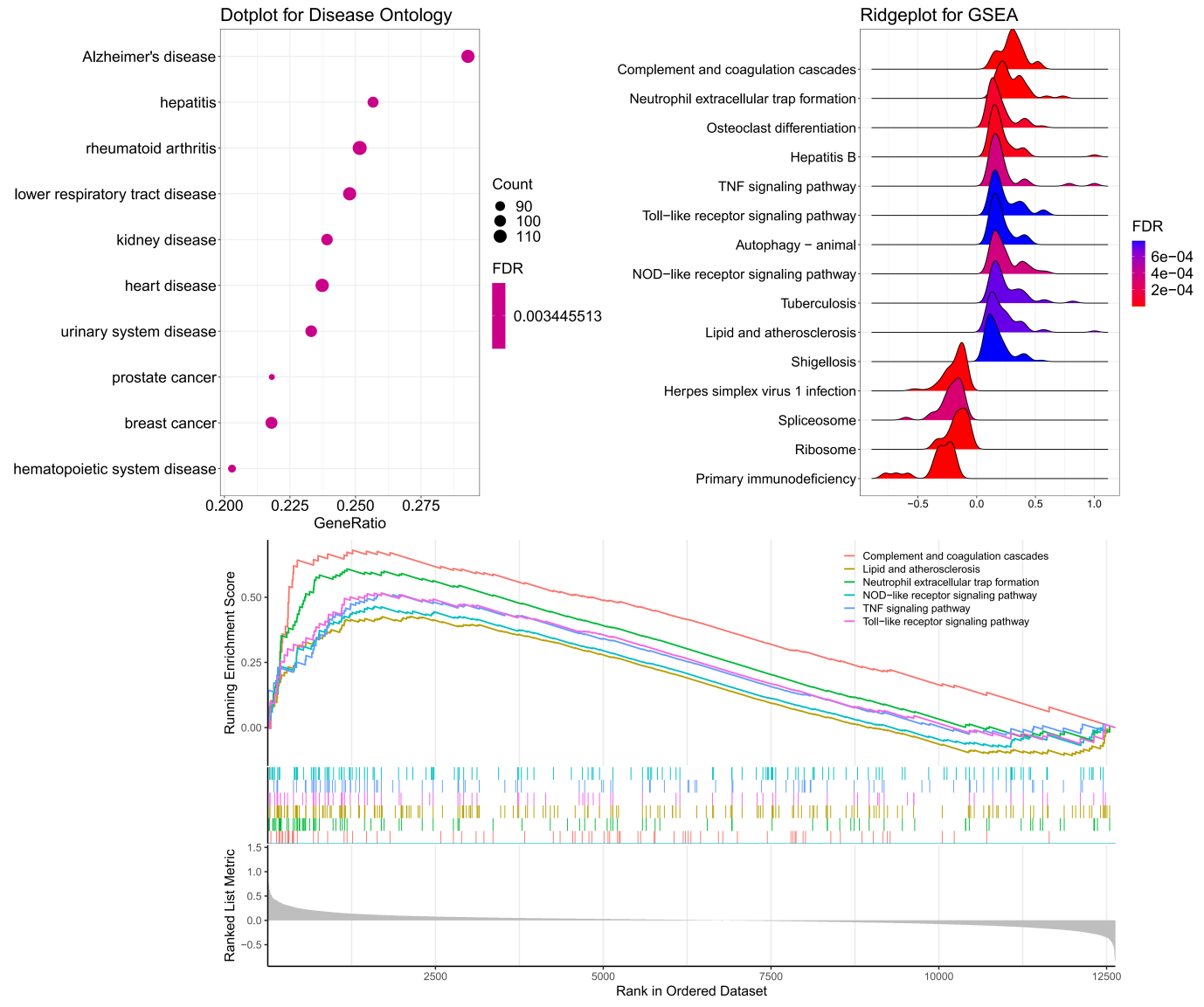


**A**

**B**

**C**

**Supplement Figure 2.** (A) DO enrichment analysis, where the circle size represents the number of DEGs under the DO term. (B) Gene set enrichment analysis, where the horizontal axis represents up-and-down regulation and the curve area represents the number of DEGs under the GSEA term. (C) Gene set enrichment analysis. P-values were determined using the Kolmogorov-Smirnov test.


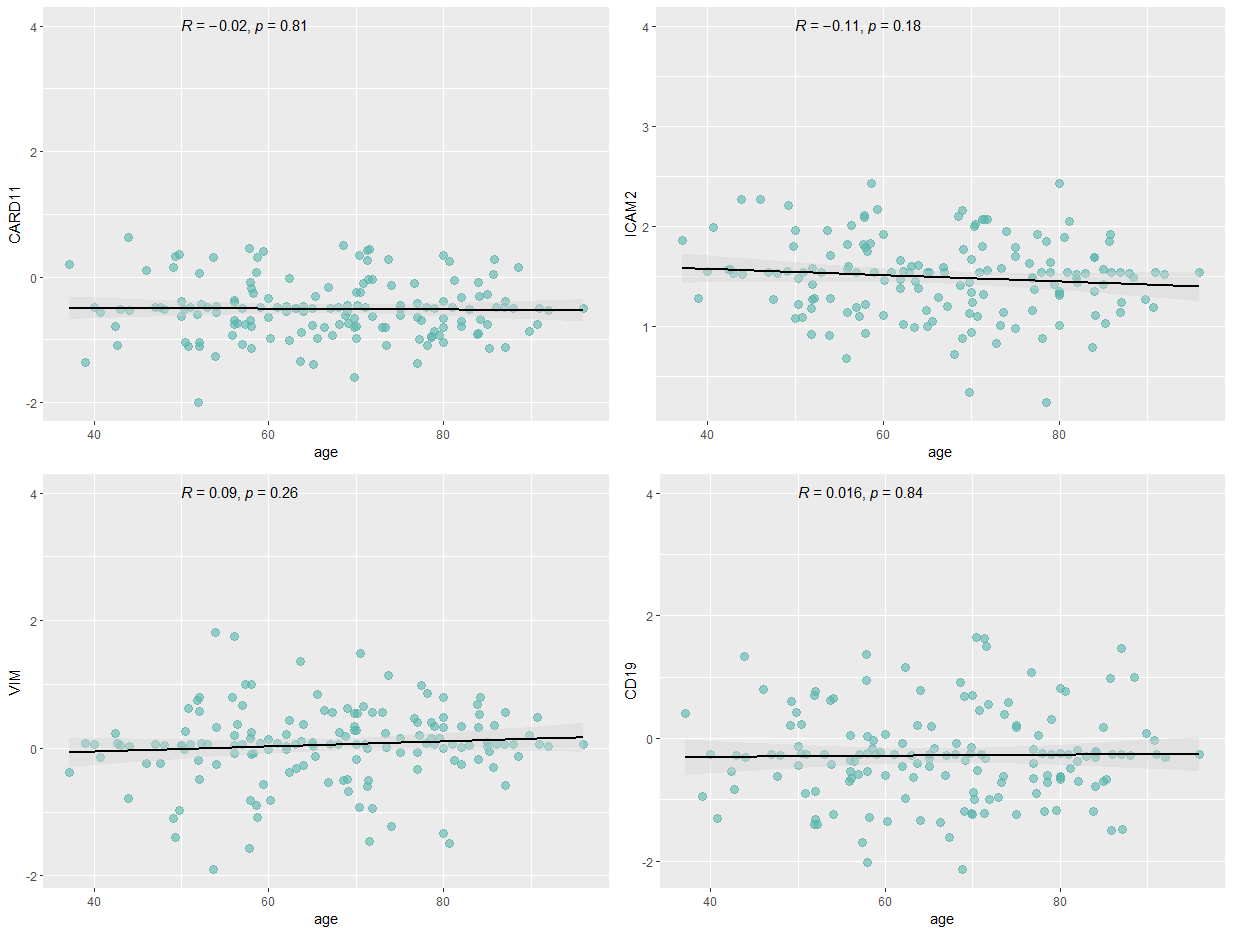


**Supplement Figure 3.** Scatter plot between expression of IRDGs and sample age.


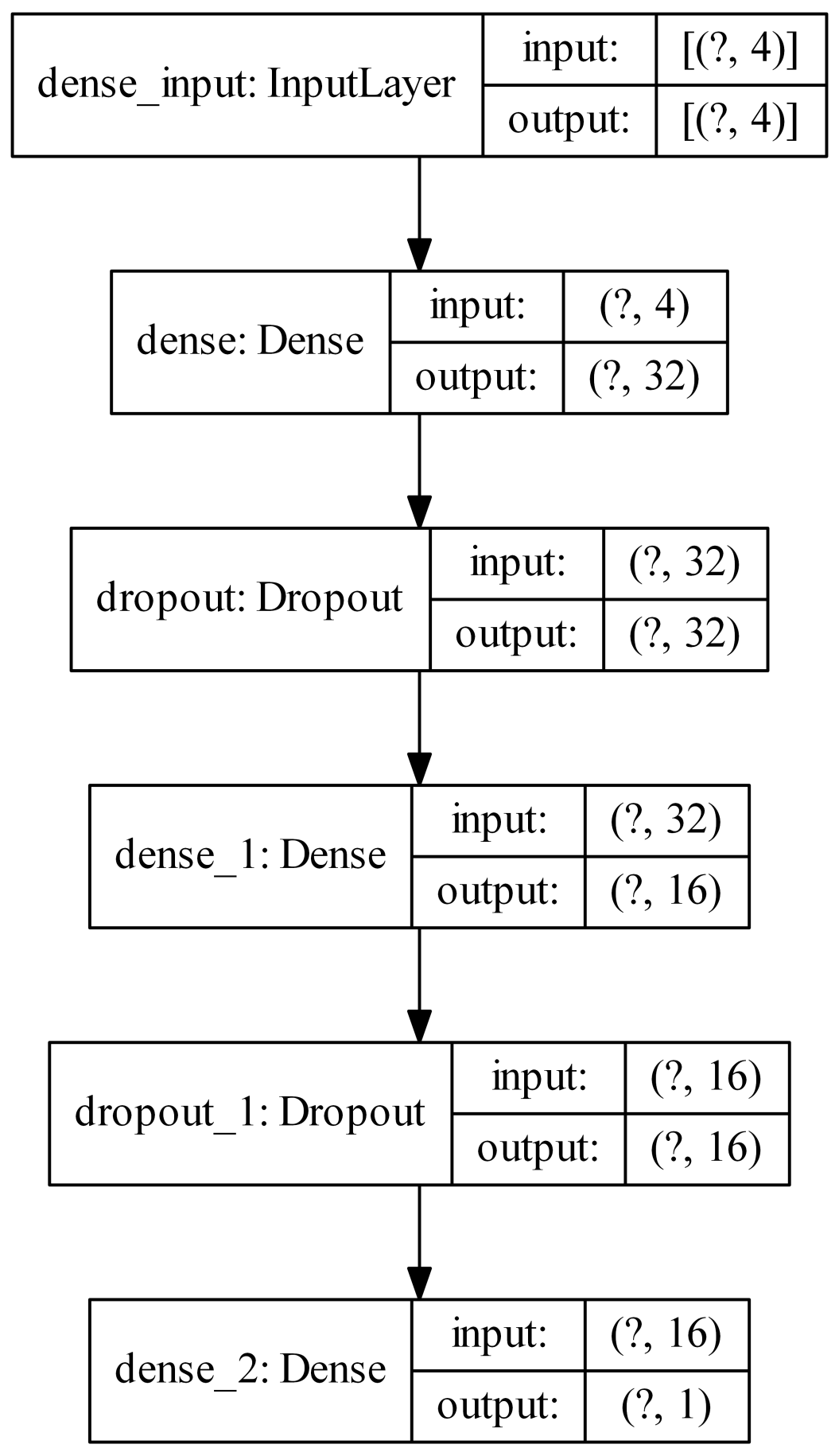


**Supplement Figure 4.** The structure diagram of neural network.


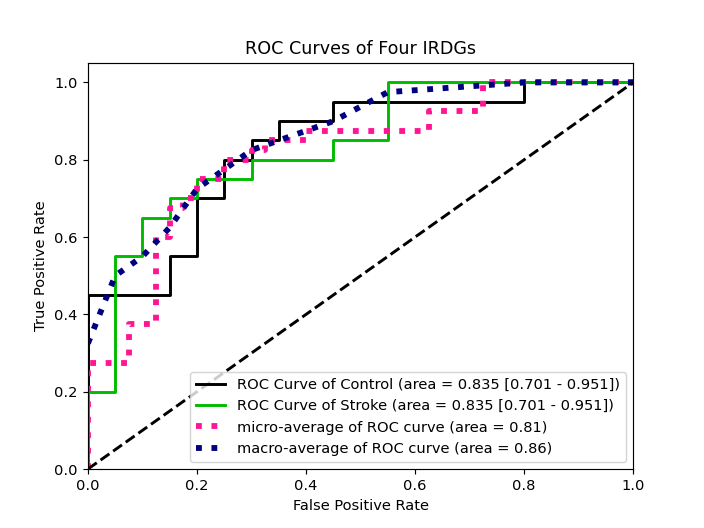


**Supplement Figure 5.** The ROC curve of the neural network diagnosis model using four selected diagnostic markers, in which the test set is GSE22255 data set.
